# Altered Surface Expression of Insulin-Degrading Enzyme on Monocytes and Lymphocytes from COVID-19 Patients Both at Diagnosis and after Hospital Discharge

**DOI:** 10.3390/ijms231911070

**Published:** 2022-09-21

**Authors:** Carlos M. González-Casimiro, Elisa Arribas-Rodríguez, Aida Fiz-López, Javier Casas, Sara Gutiérrez, Pablo Tellería, Cristina Novoa, Silvia Rojo-Rello, Eduardo Tamayo, Antonio Orduña, Carlos Dueñas, David Bernardo, German Perdomo

**Affiliations:** 1 Physiopathology of Metabolic Diseases Lab, Unidad de Excelencia Instituto de Biología y Genética Molecular (IBGM), Consejo Superior de Investigaciones Científicas (CSIC)-Universidad de Valladolid, 47003 Valladolid (UVa), Spain; 2 Mucosal Immunology Lab, Unidad de Excelencia Instituto de Biología y Genética Molecular (IBGM), Consejo Superior de Investigaciones Científicas (CSIC)-Universidad de Valladolid (UVa), 47003 Valladolid, Spain; 3 Microscopy Unit, Unidad de Excelencia Instituto de Biología y Genética Molecular (IBGM), Consejo Superior de Investigaciones Científicas (CSIC)-Universidad de Valladolid (UVa), 47003 Valladolid, Spain; 4 Internal Medicine Department, Hospital Clínico Universitario de Valladolid, 47003 Valladolid, Spain; 5 Microbiology and Immunology Unit, Hospital Clínico Universitario de Valladolid, 47003 Valladolid, Spain; 6 Department of Surgery, University of Valladolid, 47002 Valladolid, Spain; 7 Department of Anaesthesiology and Critical Care, Hospital Clínico Universitario de Valladolid, 47003 Valladolid, Spain; 8 Centro de Investigación Biomédica en Red de Enfermedades Infecciosas (CIBERINFEC), Instituto de Salud Carlos III, 28029 Madrid, Spain; 9 BioCritic, Group for Biomedical Research in Critical Care Medicine, 47005 Valladolid, Spain; 10 Centro de Investigación Biomédica en Red de Enfermedades Hepáticas y Digestivas (CIBEREHD), Instituto de Salud Carlos III, 28029 Madrid, Spain

**Keywords:** insulin-degrading enzyme, COVID-19, post-COVID-19, peripheral blood mononuclear cell, monocytes, glucose, insulin, macrophage inflammatory protein-1

## Abstract

Although the COVID-19 disease has developed into a worldwide pandemic, its pathophysiology remains to be fully understood. Insulin-degrading enzyme (IDE), a zinc-metalloprotease with a high affinity for insulin, has been found in the interactomes of multiple SARS-CoV-2 proteins. However, the relevance of IDE in the innate and adaptative immune responses elicited by circulating peripheral blood mononuclear cells is unknown. Here, we show that IDE is highly expressed on the surface of circulating monocytes, T-cells (both CD4^+^ and CD4^−^), and, to a lower extent, in B-cells from healthy controls. Notably, IDE’s surface expression was upregulated on monocytes from COVID-19 patients at diagnosis, and it was increased in more severe patients. However, IDE’s surface expression was downregulated (relative to healthy controls) 3 months after hospital discharge in all the studied immune subsets, with this effect being more pronounced in males than in females, and thus it was sex-dependent. Additionally, IDE levels in monocytes, CD4^+^ T-cells, and CD4^−^ T-cells were inversely correlated with circulating insulin levels in COVID-19 patients (both at diagnosis and after hospital discharge). Of note, high glucose and insulin levels downregulated IDE surface expression by ~30% in the monocytes isolated from healthy donors, without affecting its expression in CD4^+^ T-cells and CD4^−^ T-cells. In conclusion, our studies reveal the sex- and metabolism-dependent regulation of IDE in monocytes, suggesting that its regulation might be important for the recruitment of immune cells to the site of infection, as well as for glucometabolic control, in COVID-19 patients.

## 1. Introduction

Insulin-degrading enzyme (IDE) is a ubiquitously expressed metallo-endopeptidase characterized by a zinc-binding consensus sequence (HxxEH) that is inverted with respect to the sequence in most conventional metalloproteases (HExxH). IDE is evolutionary and ancient, with homologs and paralogs highly conserved and present in phylogenetically diverse organisms ranging from viruses to humans, suggesting that it has proteolytic and non-proteolytic functions [1,2]. The subcellular localization of IDE is primarily cytosolic, albeit it has also been reported in intracellular organelles and extracellular vesicles [1].

IDE was first characterized by its capacity to degrade insulin [3], but it can degrade, to a lower extent, a wide range of other peptide substrates including hormones (insulin and glucagon), transforming growth factor-α (TGF-α), chemokine ligand (CCL) 3, CCL4, and bradykinin (reviewed in [1,4]).

Aside from these substrates, IDE was shown to bind and degrade various viral proteins that are important for viral infection and replication. Li and colleagues were the first to report the potential role of IDE as a cellular receptor for the varicella zoster virus (VZV) [5,6]. The amino domain of IDE interacted with gE, an essential glycoprotein for VZV infection. The amino acids 24 to 71, as well as the secondary structure of gE, were important for IDE binding [7,8]. Notably, IDE catalytic activity was not necessary for binding [7]. The loss of IDE’s function inhibited VZV infection, whereas its gain-of-function resulted in increased entry and enhanced infection with both the cell-free and cell-associated virus [5]. Interestingly, Nash and colleagues, based on the sequence of the VZV gE domain, designed and generated a short peptide that effectively inhibits IDE proteolytic activity [9].

On the other hand, Hahn and colleagues demonstrated that IDE is sufficient and required for degradation of the human immunodeficiency virus (HIV-1) p6, an essential protein in the last steps of the replication cycle of the virus [10]. The proteolytic activity of IDE seems to be specific for HIV-1 since the p6 homologs of HIV-2, simian immunodeficiency virus, and p9 from an equine infectious anemia virus were insensitive to IDE degradation [10]. Interestingly, p6 is ~100-fold more efficiently degraded by IDE than insulin [10]. The competitive substrate insulin or the 6bK-mediated inhibition of IDE resulted in reduced virus replication. Likewise, HIV-1 harboring IDE-insensitive p6 mutants or a naturally occurring polymorphism of HIV-1 p6 showed reduced virus replication capacity [10,11].

In addition to VZV and HIV, IDE has been identified as a host pro-viral factor involved in the assembly/release of the hepatitis C virus (HCV) [12]. The silencing of IDE by siRNA significantly reduced the percentage of core-positive cells, level of secreted HCV RNA, and production of infectious HCV [12].

The coronavirus family is a family of positive, single-stranded RNA viruses surrounded by an envelope, and they cause respiratory and intestinal infections in humans [13]. In the last two decades, pathogenic coronaviruses including SARS-CoV (severe acute respiratory syndrome coronavirus) and SARS-CoV-2 (which causes the disease COVID-19) have caused thousands of deaths and have had an enormous impact on health systems and economies. There are viral RNA codes for four canonical structural proteins, namely, the spike (S), envelope (E), membrane (M), and nucleocapsid (N) proteins, and nonstructural proteins (Nsp) encoded by five major open reading frames (Orf) [14].

The putative role of IDE in COVID-19 infection remains unknown, albeit IDE was found in the interactomes of multiple SARS-CoV-2 Nsp and Orf proteins, such as Nsp4, Nsp12, Nsp14, Nsp15, Nsp16, and Orf3b, meaning that these proteins can interact directly or indirectly with IDE [15]. In this line of evidence, it has been reported that IDE physically interacts with Nsp4 [16]. Moreover, IDE has been identified as a COVID autoantigen [15].

To gain insights into the function of IDE on COVID-19 infection, we analyzed the expression levels of IDE in peripheral blood mononuclear cells (PBMC) isolated from healthy donors and COVID-19 patients, both at diagnosis and 3 months after hospital discharge. Our results support the concept that IDE may play a significant role in the pathophysiology of COVID-19.

## 2. Results

### 2.1. IDE Is Expressed on the Surface of Human PBMC

**Figure 1:**
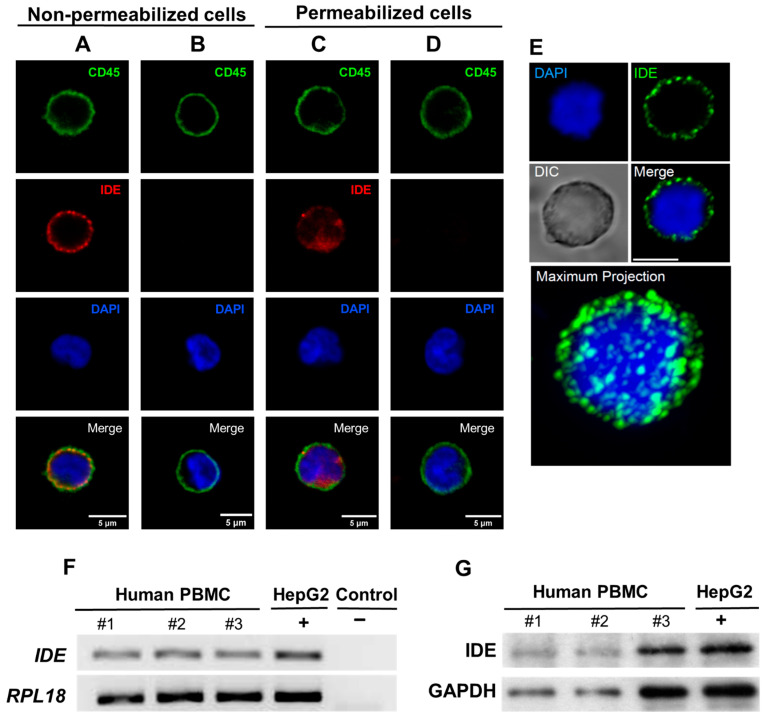
IDE expression in human peripheral blood mononuclear cells. (**A**) Representative fluorescence microscopy images of an IDE signal obtained with confocal microscopy in non-permeabilized human PBMCs. (**B**) Control experiments without the primary antibody anti-IDE in non-permeabilized cells. Calibration bar: 5 µm. (**C**) Representative image showing the specificity of IDE staining in permeabilized PBMCs. (**D**) Control experiments without the primary antibody anti-IDE in permeabilized cells. Calibration bar: 5 µm. (**E**) Representative image showing the maximal projection of a Z-stack. (**F**) IDE expression levels. mRNA was extracted from three healthy subjects (#1, #2, and #3), amplified, and resolved in an agarose gel, as described in the methods section, with human hepatocytes as a positive control (HepG2 cell line). As a negative control, PCRs were run without a cDNA template. Human *RPL18* was used as a housekeeping gene. (**G**) IDE expression levels. The total proteins were extracted from three healthy subjects (#1, #2, and #3) and subjected to Western blot analyses, as described in the methods section, with human hepatocytes as a positive control (HepG2 cell line). GAPDH was used as a loading control.

It has been described that the subcellular localization of IDE is primarily cytosolic in a wide range of cells. However, its subcellular localization in PBMCs has not been investigated. To this end, confocal microscopy was used to determine the subcellular localization of IDE in PBMCs. Surprisingly, IDE was present in the extracellular side of a plasma membrane in non-permeabilized conditions (Figure 1A,B,E and Supplementary Video S1). As expected, under permeabilizing conditions, an IDE signal was present inside the cells (Figure 1C,D). Furthermore, IDE mRNA expression (Figure 1F) and IDE protein levels (Figure 1G) were detected in human PBMCs. Taken together, these results show for the first time, to our knowledge, that IDE is localized in PBMCs at both the surface and cytosol.

### 2.2. IDE Surface Expression Varies among the Different Subsets of Human PBMCs

**Figure 2:**
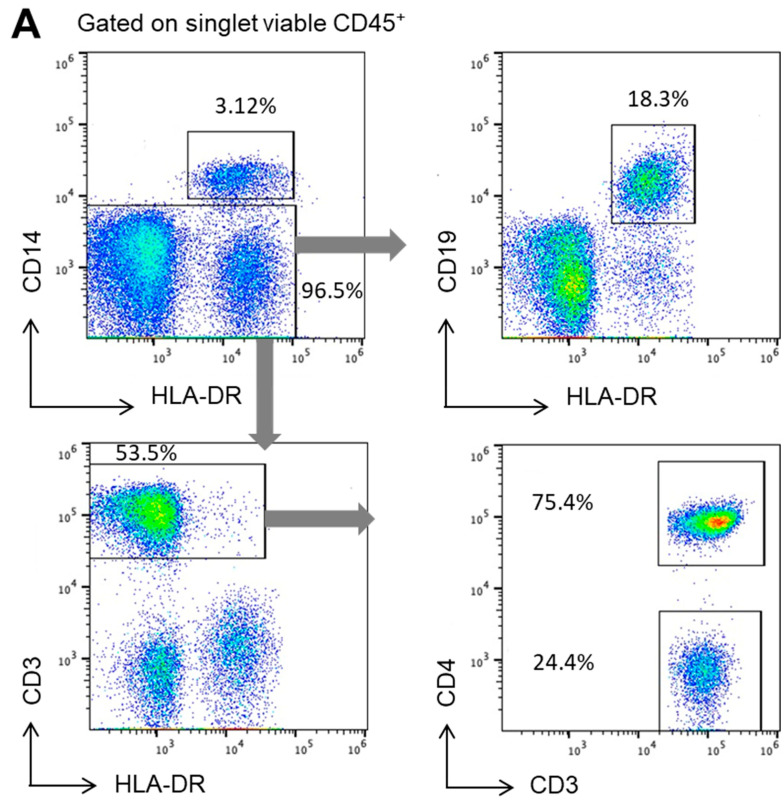

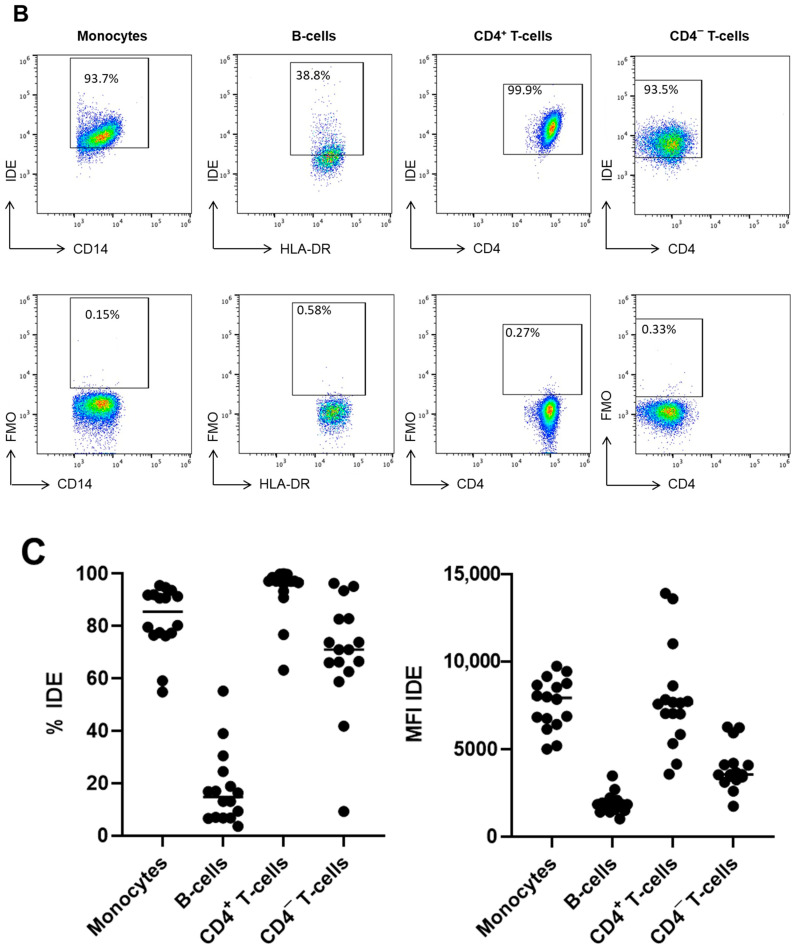
IDE surface expression on human peripheral blood mononuclear cell subsets. (**A**) Peripheral blood mononuclear cells from healthy controls were stained with several surface markers and identified within singlet viable leukocytes (CD45^+^). Monocytes were identified as HLA-DR^+^CD14^+^. Within the non-monocyte fraction, B-cells were identified as HLA-DR^+^CD19^+^ cells, while T-cells were defined by the expression of CD3 and further divided into CD4^+^ and CD4^−^ cells. (**B**) IDE expression was determined within each subset and compared to the fluorescence minus one (FMO) control. The pooled data of from several independent experiments displaying the different IDE levels of expression between the different subsets are shown in (**C**) as percentages of positive cells or as median fluorescence intensities (MFI).

Having proved that IDE is expressed on the surface of human PBMCs, we next aimed to identify whether it was expressed on all circulating PBMCs or if, on the contrary, its expression varied among different subsets. Hence, we identified by flow cytometry the most common circulating PBMC subsets, including monocytes, B-cells, CD4^+^ T-cells, and CD4^−^ T-cells (Figure 2A), and we assessed the IDE surface expression on each of them compared to their respective FMOs (Figure 2B). Hence, IDE was mainly expressed on classical CD14^+^ monocytes and T-cells (both CD4^+^ and CD4^−^), while its expression was residual on circulating B-cells (Figure 2C). Of note, the IDE surface expression on each studied subset was not influenced by the age or the gender of the pre-pandemic controls. Taken together, these results further demonstrate the presence of IDE at the surface of PBMCs. 

### 2.3. IDE Surface Expression Is Increased on Monocytes from COVID-19 Patients and Decreased in All Studied Subsets following COVID-19 Recovery

Having characterized IDE surface expression on human PBMCs from healthy controls, we next studied whether its expression was altered in patients with COVID-19, either at diagnosis or 3 months after hospital discharge (post-COVID-19).

**Figure 3:**
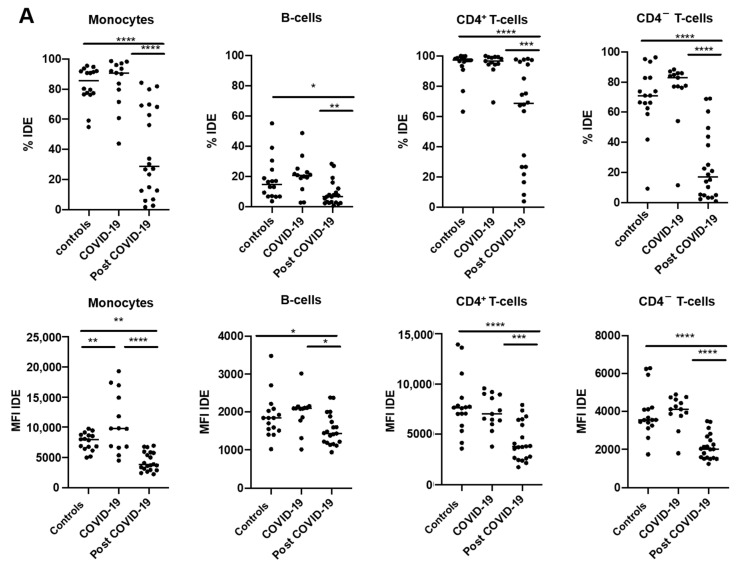

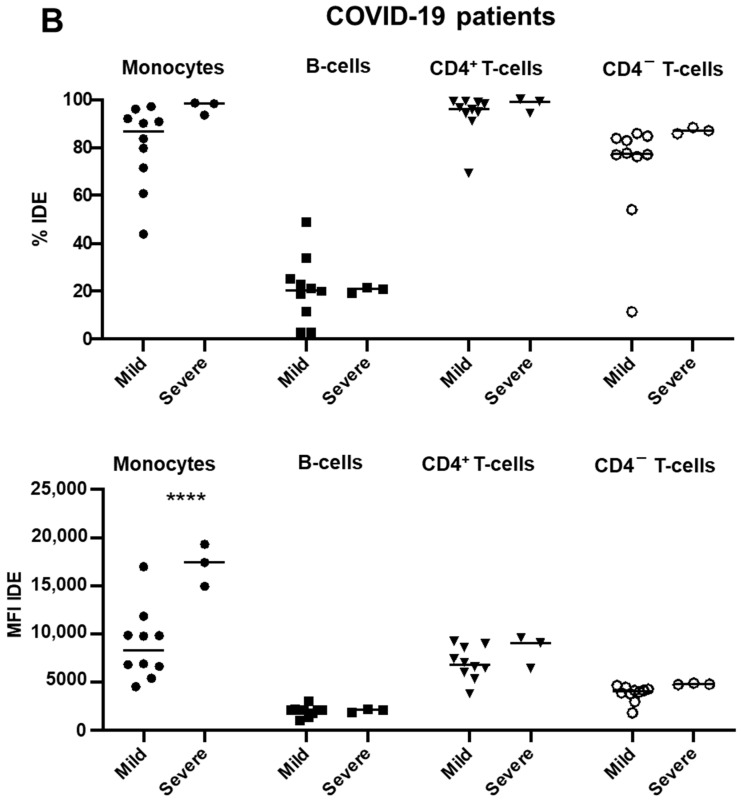

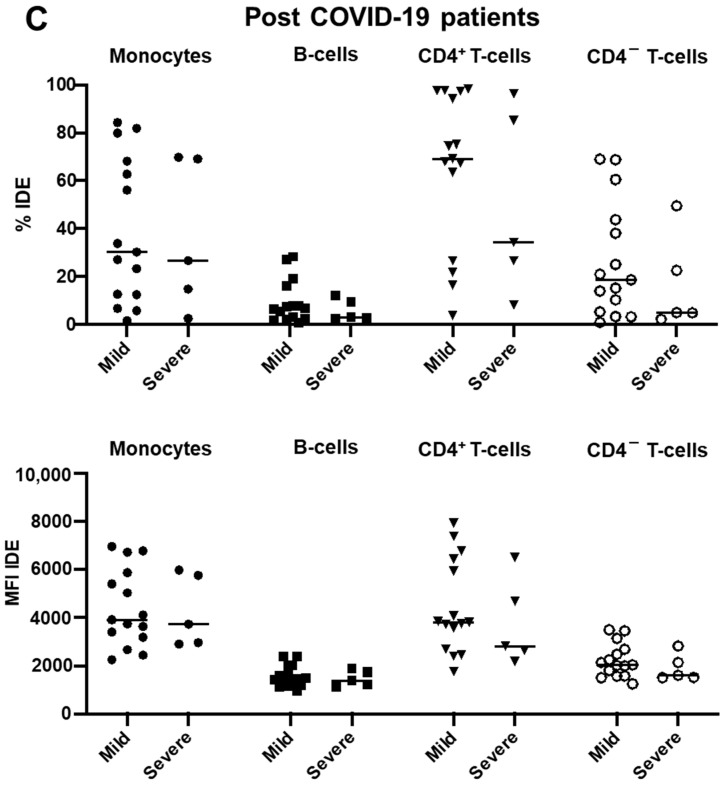
Post-COVID-19 patients have decreased surface expression of IDE on different peripheral blood mononuclear cell subsets. (**A**) The percentages and the median fluorescence indexes (MFI) of IDE expression on monocytes, B-cells, CD4^+^ T-cells, and CD4^−^ T-cells were determined as seen in Figure 2. IDE expression was determined in healthy subjects (controls), patients with COVID-19 at diagnosis (COVID-19), and patients with COVID-19 3 months after hospital discharge (post-COVID-19). The data were also analyzed based on the severity—mild or severe—in COVID-19 (**B**) and post-COVID-19 (**C**) patients. One-way ANOVA with a Sidak correction was applied in Figure 3A, while two-way ANOVA with a Sidak correction was applied in Figure 3B,C. * *p* < 0.05; ** *p* < 0.01; *** *p* < 0.001; **** *p* < 0.0001.

Although our results revealed that IDE’s MFI was increased in monocytes from COVID-19 patients at diagnosis, it was constitutively downregulated in all studied patients (both as percentages or as MFIs) 3 months after hospital discharge compared to either the healthy controls or the COVID-19 patients at diagnosis (Figure 3A).

We next studied whether IDE surface expression was influenced by the severity of the disease, revealing that those patients with worse prognoses had higher IDE MFIs on monocytes (Figure 3B). Nevertheless, the lower IDE surface expression reported in post-COVID-19 patients was independent of disease severity when the patients had been hospitalized (Figure 3C).

### 2.4. Gender Modulates IDE Surface Expression in Post-COVID-19 Patients

Having described that IDE surface expression was decreased in all studied subsets in post-COVID-19 patients (Figure 3A) and that such a decrease was independent of disease severity (Figure 3B,C), we next studied which other factors could affect IDE’s expression.

**Figure 4:**
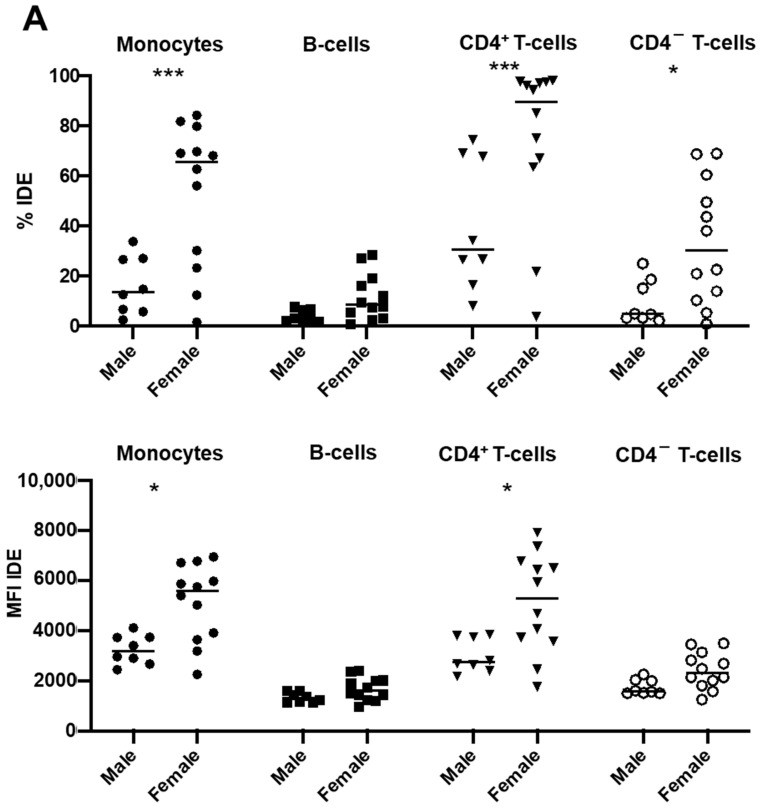

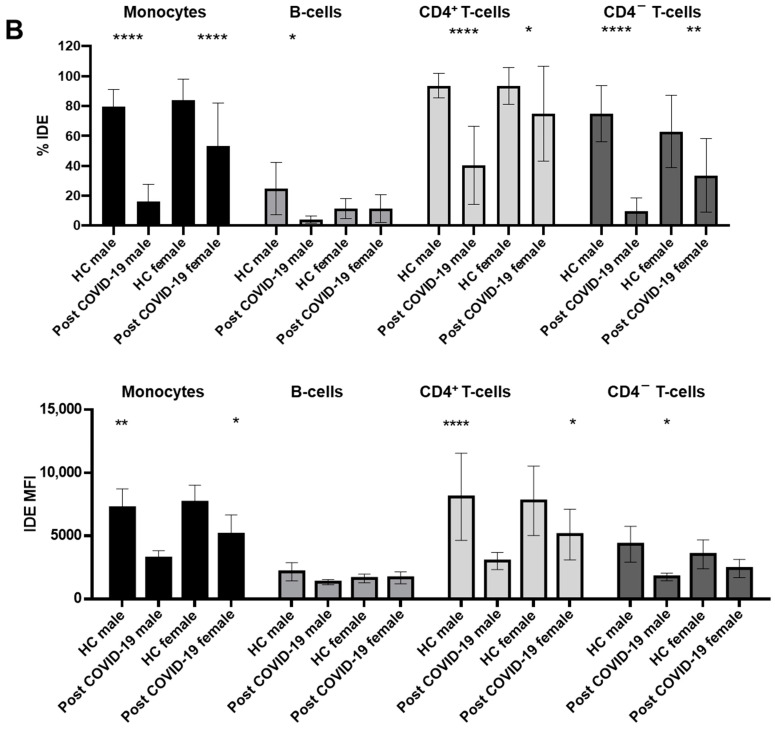
Gender influences IDE surface expression on peripheral blood mononuclear cells from post-COVID-19 patients. (**A**) The percentages and the median fluorescence indexes (MFI) of IDE expression on the monocytes, B-cells, CD4^+^ T-cells, and CD4^−^ T-cells of COVID-19 patients 3 months after hospital discharge (post-COVID-19), discriminating between males and females, was determined as seen in Figure 2. (**B**) IDE expression was also determined in male and female healthy controls (HC) compared to post-COVID-19 male and female patients, respectively. Two-way ANOVA with a Sidak correction was applied in Figure 4A, while two-way ANOVA with a Tukey correction was applied in Figure 4B. * *p* < 0.05; ** *p* < 0.01; *** *p* < 0.001; **** *p* < 0.0001.

Although age did not affect IDE surface expression in the post-COVID-19 patients, its expression was definitively affected by the gender of the patients (Figure 4A). Hence, IDE surface expression was much higher on the monocytes and CD4^+^ T-cells (both as percentages and MFIs), and partially on the CD4^−^ T-cells (only as percentages), from female post-COVID-19 patients compared to their male counterparts (Figure 4A).

Indeed, given that the female post-COVID-19 patients had higher IDE surface levels than the males, we next studied IDE’s expression in the healthy controls considering gender (Figure 4B), which revealed that there was a partial restoration of IDE expression on the PBMCs from the female post-COVID-19 patients compared to those of the males.

### 2.5. IDE Surface Expression Is Associated with Altered Metabolic Parameters

It has been reported that patients with COVID-19 have a greater prevalence of new-onset hyperglycemia, which can also be detected in post-COVID-19 patients. Furthermore, the serum hormone profile is altered in both COVID-19 and post-COVID-19 patients [17].

As shown in Table 1, post-COVID-19 patients (male and female) had higher plasma glucose and insulin levels compared to COVID-19 patients. Additionally, male patients (COVID-19 and post-COVID-19) showed higher plasma triglyceride levels as compared to female patients (Table 1).

**Figure 5:**
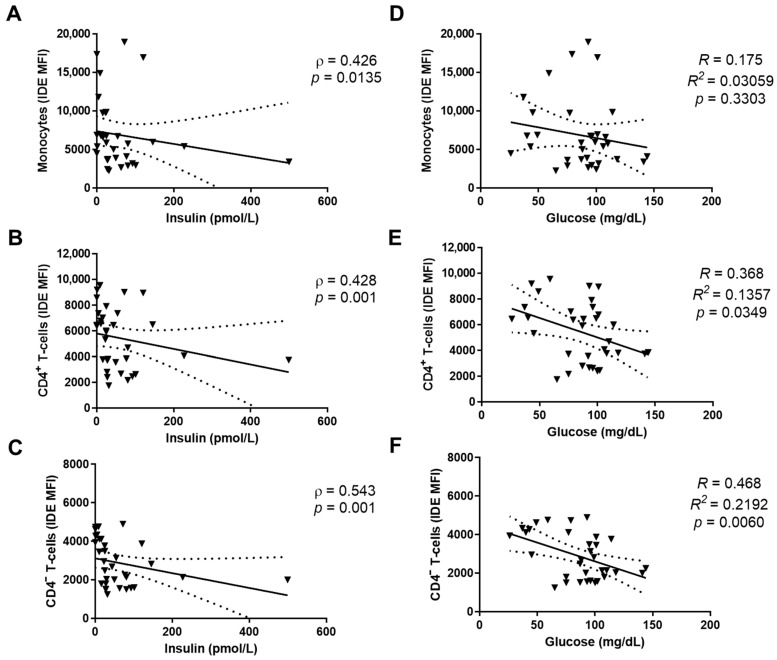
Glucose and insulin levels associated with IDE surface expression in human PBMCs from COVID-19 and post-COVID-19 patients. Bivariate analyses were performed using data from COVID-19 and post-COVID-19 patients. Correlations are shown between insulin and monocytes (**A**), CD4^+^ T-cells (**B**), and CD4^−^ T-cells (**C**). The Spearman’s correlation (ρ) and the statistical significance (*p*) are indicated in Figure 5. Correlations are shown between glucose and monocytes (**D**), CD4^+^ T-cells (**E**), and CD4^−^ T-cells (**F**). The *R^2^* and the statistical significance (*p*) are indicated.

To gain insights into the factors that regulate the IDE surface protein levels seen in PBMCs, we next performed bivariate analyses between the IDE levels and the metabolic alterations seen in patients (both COVID-19 and post-COVID-19). In monocytes, CD4^+^ T-cells, and CD4^−^ T-cells, the IDE MFIs were inversely correlated with insulin levels (Figure 5A–C). Similarly, IDE was inversely correlated with glucose levels in CD4^+^ T-cells and CD4^−^ T-cells, though no correlation was found with monocytes (Figure 5D–F).

**Figure A1:**
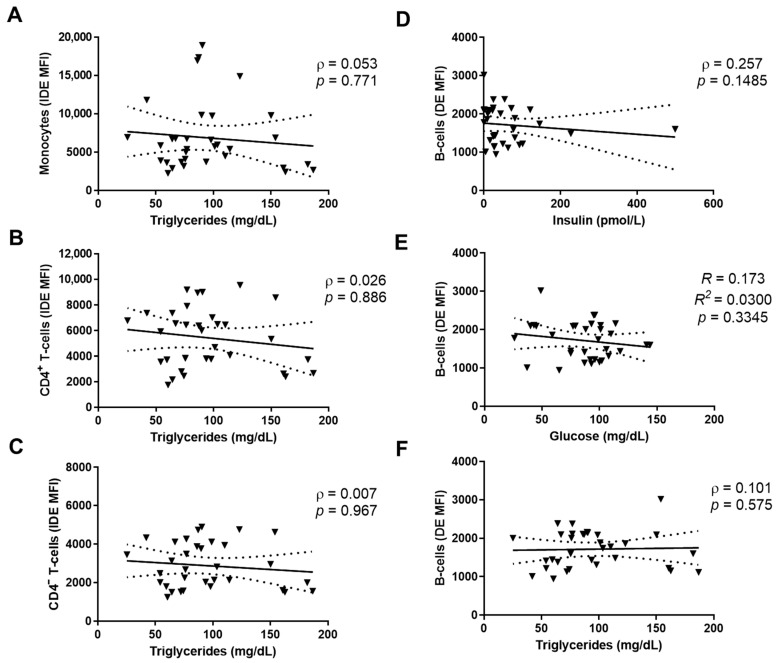
Regression analyses of the surface IDE and metabolic parameters in human PBMCs from COVID-19 and post-COVID-19 patients. Bivariate analyses were performed using data from COVID-19 and post-COVID-19 patients. Correlations between triglycerides and monocytes (**A**), CD4^+^ T-cells (**B**), and CD4^−^ T-cells (**C**) are shown. The Spearman’s correlation (ρ) and statistical significance (*p*) are indicated. Correlations between B-cells and insulin (**D**), glucose (**E**), and triglycerides (**F**) are shown. The *R^2^* or ρ coefficients and the statistical significance (*p*) are indicated.

Finally, no correlations were observed between the IDE surface levels and triglyceride levels in monocytes, CD4^+^ T-cells, and CD4^−^ T-cells (Appendix A, Figure A1A–C). Likewise, the surface IDE levels in B-cells did not correlate with insulin, glucose, or triglyceride levels (Appendix A, Figure A1D–F).

### 2.6. Glucose and Insulin Reduce Surface IDE Expression on Circulating Classical Monocytes

Having proved an association between the surface IDE levels and the metabolic perturbations in the PBMCs from COVID-19 and post-COVID-19 patients, we sought to investigate whether insulin and glucose could directly modulate IDE expression on circulating human PBMCs.

**Figure 6:**
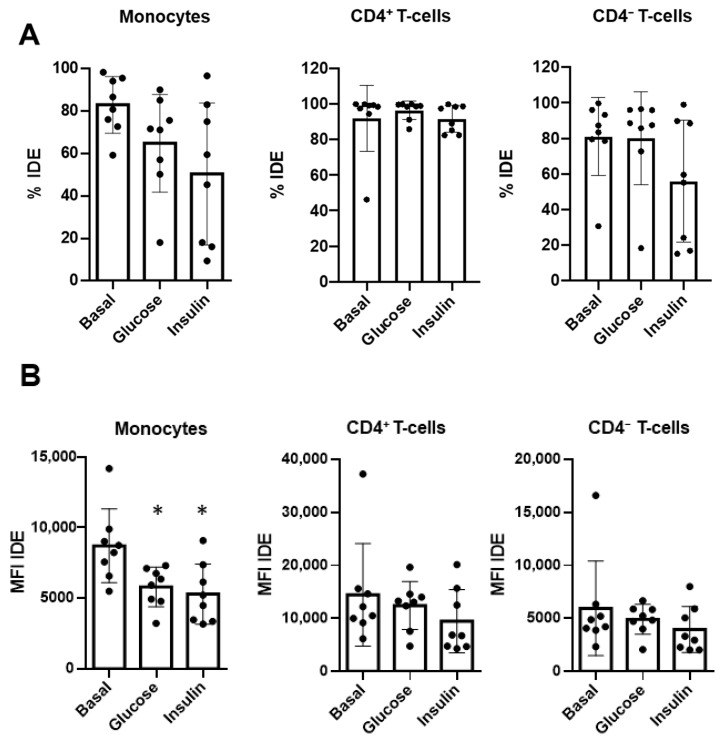
IDE surface expression on monocytes is modulated by glucose and insulin. Peripheral blood mononuclear cells from healthy controls were ex vivo cultured in resting conditions (basal), in the presence of glucose (22 mM), or in the presence of insulin (2500 pmol/L). IDE expression, determined both as a percentage (**A**) and as the median fluorescence index (MFI) (**B**) on monocytes, B-cells CD4^+^ T-cells, and CD4^−^ T-cells, was assessed as in Figure 2. One-way ANOVA repeated measures with a Dunnet correction were applied following ad hoc comparisons to the basal culture. * *p* < 0.05.

Hence, the total PBMCs from healthy controls were ex vivo challenged by the presence of high levels of glucose (2500 pmol/L) or insulin (22 mM). As shown in Figure 6, IDE surface expression (defined by MFI) on classical monocytes was downregulated by both factors compared to the control cells. In contrast, IDE expression remained unchanged in CD4^+^ T-cells and CD4^−^ T-cells (Figure 6A,B). Thus, our results demonstrate a cause–effect relationship between both glucose and insulin and IDE surface expression on human monocytes.

## 3. Discussion

Here, using two different methodological approaches (confocal microscopy and flow cytometry), we have demonstrated, for the first time to our knowledge, that IDE is expressed on the surface of monocytes, B-cells, CD4^+^ T-cells, and CD4^−^ T-cells. In addition, IDE expression was downregulated in COVID-19 patients 3 months after hospital discharge, revealing a sex- and metabolic-dependent regulation of IDE in monocytes, suggesting that its regulation might be important for the recruitment of immune cells to the site of infection and glucometabolic control in COVID-19 patients.

The subcellular localization of IDE has been extensively studied, but its extracellular localization remains controversial. Although IDE has been classically localized to the cytosol, early literature referred to “cell surface- or membrane-associated” IDE [18]. More recently, it has been proposed that IDE can also be biotinylated on the plasma membrane of non-neural cells (Chinese Hamster Ovary; CHO) [19] and differentiated neuronal cells (rat PC12) [20]. It is intriguing how a protein with no transmembrane domain or canonical site for membrane anchorage predicted from the primary structure of IDE can be localized at the extracellular side of the plasma membrane. A plausible explanation may lie in the presence of a polyanion binding site in the structure of human IDE, which has been described to interact with membrane-bound phosphatidylinositol phosphates [21]. Additionally, IDE exhibits a putative site for myristylation at Gly51 (score 0.98; Expasy website, the SIB Swiss Institute of Bioinformatics). Protein N-myristylation is a cotranslational lipidic modification process that occurs in the cytoplasm of cells, which is relevant for targeting proteins to plasma membranes and in regulating immune functions [22,23]. Finally, IDE might interact with other protein(s) at the outer side of the plasma membrane. Clearly, further research is required to unveil how IDE attaches to the surface of human monocytes, CD4^+^ T-cells, and CD4^−^ T-cells, and, to a lower extent, B-cells.

Additionally, the potential export/secretion mechanism of IDE is still obscure because the lack of a signal peptide argues against IDE’s association to the extracellular side of a plasma membrane. In this line of evidence, Corraliza-Gómez and collaborators have shown that stimulated microglia allow IDE to be exported outside of cells in small extracellular vesicles that have arisen from multivesicular bodies, albeit, in their study, it was associated with plasma membranes exclusively at the cytoplasmic side [2]. Thus, it is plausible to hypothesize that IDE might be secreted from monocytes and lymphocytes via non-conventional secretory pathways. In support of this view, interleukin-1β is secreted by activated human monocytes via a pathway of secretion that is different from the classical endoplasmic reticulum–Golgi route, possibly involving the translocation of intracellular membranes, and this process is increased by stress conditions [24]. Additionally, there are examples in the literature of proteins lacking conventional signal peptides, such as the IDE paralog metalloprotease N-arginine dibasic convertase (a.k.a., nardilysine) [24,25,26], the metalloendoprotease EP24.15 [27], or basic fibroblast growth factor [28], which are localized outside of cells. Further studies are needed to elucidate how IDE is exported to the extracellular side of a plasma membrane in monocytes and lymphocytes.

In any case, the presence of IDE at the cell surface in monocytes and lymphocytes opens new perspectives concerning its biological function. As a promiscuous metalloprotease, IDE can degrade a wide range of substrates, including CCL3 (i.e., macrophage inflammatory protein-1α (MIP-1α)) and CCL4 (MIP-1β), which are small peptides required for the recruitment of immune cells to the site of infection or inflammation [29,30]. Recently, it has been reported that CCL3 and CCL4 are significantly upregulated at later stages of the disease in patients with severe COVID-19. The expression patterns of CCL3 and CCL4 were primarily defined by disease-related CD169^−^ classical monocytes (M4-M6) in combination with CCL3, CCL4, and CCL23 [31]. Further studies are warranted to determine surface IDE levels in different subsets of disease-related classical monocytes and its relevance in regulating CCL3 and CCL4 levels in COVID-19 patients.

Recently, Montefusco and collaborators described a greater prevalence of new-onset hyperglycemia in patients hospitalized for COVID-19. Of note, these patients did not have a pre-existing history or diagnosis of diabetes mellitus, but their clinical outcomes were the poorest, with more requirements for ventilation, longer lengths of hospitalization, and need for the intensive care unit [17]. Of note, glycemic alterations (elevated levels of glucose and insulin) persisted in some of those who had recovered from COVID-19 [17]. In this work, we found significant correlations between altered glucometabolic control and the surface expression of IDE in monocytes and lymphocytes in COVID-19 and post-COVID-19 patients. Furthermore, high levels of glucose or insulin decreased the expression of IDE on the surface of monocytes in healthy donors. These data support further investigation on the role of IDE in metabolic abnormalities in the context of COVID-19 and post-COVID-19 patients.

In conclusion, we have reported variations in IDE on the surface of myeloid cells in the context of COVID-19 infection which are sex-dependent and persist 3 months after hospital discharge. Our results open new avenues for investigating the role of IDE in the pathogeny of COVID-19, with particular emphasis on the recruitment of immune cells to the site of infection or inflammation, as well as on glucometabolic control.

## 4. Materials and Methods

### 4.1. Patient Recruitment

COVID-19 patients attending the Accident and Emergency Unit at Hospital Clínico Universitario (Valladolid, Spain) were recruited during the months of March and April in 2020. All COVID-19 patients had a PCR-confirmed diagnosis. Blood samples were obtained at patient admission. Samples from post-COVID-19 patients were obtained 3 months after hospital discharge. The pre-pandemic controls included in this study were provided by the Spanish National DNA Bank Carlos III of the University of Salamanca (PT17/0015/0044) and integrated in the Spanish National Biobanks Network. Patient demographics (including age, gender, and days in hospital, if applicable) are shown in Table 2. The COVID-19 patients (either at diagnosis or 3 months after hospital discharge) were also classified, based on severity, as mild (no hospitalization or less than 10 days in the hospital) or severe (more than 10 days of stay at the hospital and/or exitus). For functional experiments, blood samples from 8 healthy controls with unknown autoimmune, inflammatory, or malignant disease were recruited. All patients provided written informed consent following ethics approval by the local ethics committee from East Valladolid (Comité Ético de Investigación con Medicamentos (CEIM) de Valladolid Este; PI-20-1716). All methods were performed in accordance with the relevant guidelines and regulations.

### 4.2. Biological Material

Blood samples from the COVID-19 and post-COVID-19 patients were placed in heparin-lithium tubes and centrifuged over a Ficoll-Paque PLUS (Amersham Biosences, Chalfnt St Giles, UK) to obtain the plasma (which was immediately aliquoted at −80 °C until used) and PBMCs. The PBMCs were further washed in RPMI-1640 and cryopreserved at −80 °C in freezing medium (90% fetal calf serum and 10% DMSO) until needed. Plasma and PBMCs from the pre-pandemic controls were directly provided by the Spanish National Biobanks Network. Prior to use, the PBMCs were partially defrosted in a warm bath at 37 °C and subsequently washed in complete medium (Dutch modified RPMI 1640 (Sigma-Aldrich, Dorset, UK) containing 100 µg/mL penicillin/streptomycin, 2 mM L-glutamine, 50 µg/mL gentamicine (Sigma-Aldrich, Dorset, UK), and 10% fetal calf serum (TCS cellworks, Buckingham, UK)) before performing flow cytometry.

### 4.3. Antibody Labelling

The PBMCs were washed twice in PBS containing 1 mM EDTA and 0.02% sodium azide (FACS buffer) before being stained with monoclonal or polyclonal (anti-IDE) antibodies and characterized by flow cytometry. In all cases, a live/dead fixable near-IR dead cell stain kit (Molecular Probes, Eugene, OR, USA) was added to the cells prior to performing antibody staining, allowing the exclusion of dead cells from the analysis. Table 3 shows the specificity, source, clone, fluorochrome, and manufacturer of the antibodies used. The cells were labelled in FACS buffer on ice and stored in the dark for 20 min following Fc block incubation (Becton Dickinson, Madrid, Spain). For the assessment of IDE, a secondary antibody was used. In all cases, cells were further washed in FACS buffer, fixed with 2% paraformaldehyde in FACS buffer for 10 min on ice, and washed again in FACS buffer before they were stored at 4 °C prior to acquisition on the flow cytometer.

### 4.4. Flow Cytometry and Data Analysis

The cells were acquired on a Galios cytometer (Beckman Coulter, Brea, CA, USA). In all cases, the results were analyzed using FlowJow (version 10.1, Ashland, OR, USA). All cells were analyzed within singlet viable CD45^+^ cells. Positive and negative gatings were set by the fluorescence minus one (FMO) method.

### 4.5. Plasma Biochemistry and Insulin Assessments

The plasma glucose and triglyceride levels were assessed as previously described [32]. The plasma insulin levels were assessed using human enzyme-linked immunosorbent (ELISA) assay following the manufacturer’s instructions (Mercodia, Uppsala, Sweden).

### 4.6. Cell Culture

For ex-vivo cultures, freshly obtained PBMCs [33] were seeded in 6-well plates at a density of 200,000 cells/mL in RPMI-1640 (w/o glucose supplemented with 10% FBS, 100 U/mL penicillin, and 100 μg/mL streptomycin) for 4 h. Then, the cells were washed in warm PBS and pre-incubated in RPMI-1640 medium without FBS but supplemented with 2 mM glucose (basal). To determine the effects of metabolic milieu on the surface expression of IDE, the cells were challenged with high insulin levels (2500 pmol/L insulin plus 2 mM glucose) or with high glucose concentrations (22 mM). After 18 h, the cells were carefully harvested, washed, and stained as described above, before flow cytometry analyses. HepG2 cells (ATCC, Manassas, VA, USA) were seeded in 6-well plates at a density of 150,000 cells/mL in MEM supplemented with 10% FBS, 100 U/mL penicillin, and 100 μg/mL streptomycin.

### 4.7. Confocal Microscopy

For the surface staining of extracellular IDE, the PBMCs were incubated in Fc block (564219, Becton Dickinson, Madrid, Spain) solution in FACS buffer with 2% normal goat serum at room temperature (RT) in the dark for 10 min. After washing in FACS buffer, the cells were stained with rabbit anti-IDE antibody (dilution 1:400) (AB9210, Millipore, Temecula, MA, USA) for 20 min in the dark at 4 °C. After washing, the cells were incubated with an Alexa Fluor^®^ 488-conjugated F(ab’)2-goat anti-rabbit IgG dilution (1:200) (A11070, Invitrogen, Waltham, MA, USA) for 20 min in the dark at 4 °C. Then, the cells were washed and fixed with 2% paraformaldehyde for 10 min in the dark at 4 °C. After surface staining, the cells were placed with 100 µL of FACS buffer on German Glass poly-L-lysine-treated round coverslips and incubated for 1 h in the dark at RT, allowing them to attach to the glass. Finally, coverslips were mounted in appropriate microscope slides with Fluoroshield™ mounting media with DAPI (F6057, Sigma-Aldrich, St. Louis, MO, USA) for nuclei counterstaining.

For experiments with permeabilized cells, PBMCs attached to poly-L-lysine-treated glass coverslips were fixed with 2% (*v*/*v*) FACS-buffered paraformaldehyde for 10 min in the dark at 4 °C. Following being washed in FACS buffer, the cells were permeabilized in 0.1% (*v*/*v*) Triton X-100 in FACS buffer for 5 min. Then, the cells were blocked with 2% (*v*/*v*) FBS in Fc block solution for 30 min. at RT, followed by incubation with primary antibody (anti-IDE; 1:2000) or FACS buffer with 2% (*v*/*v*) FBS (control) for 30 min. in the dark at 4 °C. FITC-conjugated donkey anti-rabbit IgG (#406403, BioLegend, San Diego, CA, USA) was used as a secondary antibody (dilution 1:50). After washing, the samples were mounted with Fluoroshield™ and DAPI (F6057, Sigma-Aldrich, St. Louis, MO, USA).

All images were captured with a Leica (Madrid, Spain) confocal system TCS SP5X inverted microscope with an HCS Plan Apo CS 63X/1.4 NA oil immersion lens. Leica Application Suite Advanced Fluorescence software was used for the capture, confocal z-stacks images were deconvolved using an ImageJ (version 1.53, Bethesda, MD, USA) parallel iterative plugin with the Wiener Filter Preconditioned Landweber (WPL) method after calculating the experimental setup point spread function (PSF) using sub-resolution (0.17 μm) fluorescent beads.

### 4.8. PCR and Electrophoresis in Agarose Gel

The total RNAs were purified from human PBMCs isolated by ficoll gradient from 5 mL of whole blood from each of the three healthy donors and from ~50,000 cells of human hepatocytes (HepG2 cell line) as the positive control. TRIzol™ Reagent (Invitrogen™, Waltham, MA, USA) was used according to the manufacturer’s instructions. Quantification of the total RNA was performed measuring ultraviolet absorbance in a NanoDrop^®^ ND-1000 (Controltecnica, Spain) full-spectrum spectrophotometer. The removal of any potential genomic DNA contamination was achieved by treating the samples with the RapidOut DNA Removal Kit (Thermo Scientific™, Waltham, MA, USA). Complementary DNA (cDNA) was synthesized using the iScript™ cDNA Synthesis Kit (Bio-Rad™, Madrid, Spain) according to the manufacturer’s instructions.

The mRNA levels of the target and housekeeping genes were determined by real-time quantitative PCR (qPCR) with TaqMan™ assays on a LightCycler^®^ 480 System (Roche, Basel, Switzerland). The qPCRs were carried out on equal cDNA amounts, in triplicate, for each sample using a Maxima Probe qPCR Master Mix (Thermo Scientific™, Waltham, MA, USA). The human ribosomal protein L18 (*RPL18*) was used as a housekeeping gene. The following primers were used for *RPL18*: forward: 5′-AACTGATGATGtGCGGGTTC-3′; reverse: 5′-CAGCTGGTCGAAAGTGAGG-3′; and probe: 5′-FAM-CTGAAGGTATGTGCACTGCGCGTGA-BHQ1-3′. For IDE, the TaqMan^®^ Gene Expression assay (Applied Biosystems, Waltham, MA, USA) reference was Hs00610452_m1.

In order to compare the size by the apparent molecular weights of the amplification products from the PBMCs and HepG2 samples, after qPCR, amplicons from the target and housekeeping cDNAs were dyed with DNA loading dye (Thermo Scientific™, Waltham, MA, USA) and resolved by wet electrophoresis in a 2% agarose gel stained with SYBR^®^ Safe nucleic acid gel stain (Invitrogen™, Waltham, MA, USA) and read under ultraviolet light.

### 4.9. Western Blot Analyses

Western blot analyses were performed on PBMCs isolated by ficoll gradient from 5 mL of whole blood from each of three healthy donors and from ~50,000 cells of the HepG2 cells as the positive control. The cells were disolved in 30 µL ice-cold lysis buffer (125 mmol/L Tris-HCl pH 6.8, 2% (*w*/*v*) SDS and 1 mmol/L dithiothreitol) supplemented with protease and phosphatase cocktail inhibitors (Sigma, St. Louis, MO, USA) and 1 mmol/L phenylmethylsulphonyl fluoride (PMSF; Merck Life Science, Madrid, Spain). Then, the lysates were sonicated for 3 min on ice and centrifuged at 18,500× *g* for 10 min at 4 °C to separate and discard the insoluble materials. The supernatants were kept and an aliquot was used for quantifying the protein content using the Pierce BCA proteins assay kit (ThermoFisher^TM^, Waltham, MA, USA).

A total of 20 µg of solubilized proteins were resolved by 10% SDS-PAGE and then electrotransferred onto polyvinylidene difluoride (Immobilon-P, Millipore, MA, USA) filters for immunoblotting by conventional means. After probing with specific rabbit polyclonal anti-IDE (1:40,000; #AB9210, Millipore, MA, USA) and mouse anti-glyceraldehyde-3-phosphate dehydrogenase (GAPDH) (1:40,000; #MAB374, Millipore, MA, USA), the membranes were incubated with horseradish peroxidase-conjugated secondary antibodies, donkey anti-Rabbit IgG (H+L)) (1:20,000; #711-035-152; Jackson Immunoresearch, West Grove, PA, USA), and sheep anti-mouse IgG (1:5000; NA9310; Amersham, Piscataway, NJ, USA), respectively.

The signals were detected by chemiluminescence (Clarity Western ECL Substrate, Bio-Rad™, Madrid, Spain) and exposure to X-ray film to produce bands within the linear range.

### 4.10. Statistical Analysis

Statistical analysis was performed using Prism v. 6.0 (GraphPad Software, Inc., San Diego, CA, USA). The normality of the distribution of the data was checked with the Kolmogorov–Smirnov test. The data are presented as the means ± SEM. Comparisons between more than two groups were completed using one- or two-way ANOVA (with or without repeated measures) and subsequent Tukey, Sidak, or Dunnet pots-hoc corrections were applied as detailed in each figure legend. Bivariate analyses were performed using the Pearson’s correlation coefficient (R) for normal data distribution or nonparametric Spearman’s correlation (ρ). The level of significance was fixed at *p* < 0.05 in all cases.

## Figures and Tables

**Table 1:** Plasma metabolites and hormones in COVID-19 and post-COVID-19 patients.

	COVID-19		Post-COVID-19	
	Female	Male	Female	Male
Number of patients (n)	13		20	
	5	8	12	8
Glucose (mg/dL)	67.0 ± 8.0		98.5 ± 4.2 *	
	64.2 ± 13.5	68.8 ± 9.8	93.0 ± 3.6 *	106.8 ± 8.3 *
Insulin (pmol/L)	22.7 ± 9.4		85.9 ± 23.9 *	
	20.8 ± 11.6	24.0 ± 13.4	67.7 ± 17.3 *	113.2 ± 52.5 *
Cholesterol (mg/dL)	102.2 ± 6.0		125.9 ± 3.9 *	
	107.7 ± 8.6	98.8 ± 7.9	124.6 ± 4.6	127.8 ± 6.9 *
Triglycerides (mg/dL)	98.0 ± 8.3		93.1 ± 10.0	
	74.8 ± 8.8	112.4 ± 9.1 ^$^	72.0 ± 6.9	124.7 ± 17.5 ^$^

Data are the means ± SEM. * *p* < 0.05 vs. COVID-19 patients by ANOVA. ^$^ *p* < 0.05 vs. female by ANOVA.

**Table 2:** Gender, age, days spent in hospital (if applicable), and severity of the pre-pandemic controls, COVID-19 patients at diagnosis, and post-COVID-19 patients 3 months after hospital discharged recruited in this study.

	Gender	Age	Days in Hospital	Severity
Control.1	Male	46	n/a	n/a
Control.2	Male	45	n/a	n/a
Control.3	Male	60	n/a	n/a
Control.4	Male	64	n/a	n/a
Control.5	Male	62	n/a	n/a
Control.6	Male	66	n/a	n/a
Control.7	Male	65	n/a	n/a
Control.8	Female	46	n/a	n/a
Control.9	Female	60	n/a	n/a
Control.10	Female	41	n/a	n/a
Control.11	Female	63	n/a	n/a
Control.12	Female	45	n/a	n/a
Control.13	Female	63	n/a	n/a
Control.14	Female	45	n/a	n/a
Control.15	Female	61	n/a	n/a
COVID-19.1	Male	47	6	mild
COVID-19.2	Male	32	3	mild
COVID-19.3	Male	75	4	mild
COVID-19.4	Male	62	4	mild
COVID-19.5	Male	61	4	mild
COVID-19.6	Male	33	0	mild
COVID-19.7	Male	89	Exitus	severe
COVID-19.8	Male	83	Exitus	severe
COVID-19.9	Female	62	0	mild
COVID-19.10	Female	35	0	mild
COVID-19.11	Female	52	0	mild
COVID-19.12	Female	82	0	mild
COVID-19.13	Female	88	40	severe
Post-COVID-19.1	Male	62	6	mild
Post-COVID-19.2	Male	75	4	mild
Post-COVID-19.3	Male	51	6	mild
Post-COVID-19.4	Male	47	6	mild
Post-COVID-19.5	Male	75	4	mild
Post-COVID-19.6	Male	41	23	severe
Post-COVID-19.7	Male	75	15	severe
Post-COVID-19.8	Male	64	12	severe
Post-COVID-19.9	Female	64	8	mild
Post-COVID-19.10	Female	79	2	mild
Post-COVID-19.11	Female	91	7	mild
Post-COVID-19.12	Female	59	8	mild
Post-COVID-19.13	Female	62	5	mild
Post-COVID-19.14	Female	58	5	mild
Post-COVID-19.15	Female	42	3	mild
Post-COVID-19.16	Female	56	4	mild
Post-COVID-19.17	Female	59	3	mild
Post-COVID-19.18	Female	59	7	mild
Post-COVID-19.19	Female	81	11	severe
Post-COVID-19.20	Female	51	16	severe

**Table 3:** Specificity, source, clone, conjugate, and manufacturer of the antibodies used in this study.

Specificity	Source	Conjugate	Clone	Manufacturer
CD3	Mouse	APC	HIT3a	Biolegend
CD14	Mouse	Alexa700	61D3	eBioscience
CD19	Mouse	PE-Cy5	HIB19	Becton Dickinson
CD45	Mouse	PE-Cy7	HI30	Becton Dickinson
CD45	Mouse	FITC	304038	Biolegend
HLA-DR	Mouse	BV510	L243	Biolegend
IDE	Rabbit	n/a	AB9210	Sigma-Aldrich
Rabbit IgG	Donkey	FITC	Poly4064	Biolegend
Rabbit IgG	Goat	Alexa488	A11070	Invitrogen
Rabbit IgG	Goat	Alexa594	A11012	Invitrogen
GAPDH	Mouse	n/a	MAB374	Sigma-Aldrich
Rabbit IgG	Donkey	HRP	711-035-152	Jackson Immunoresearch
Mouse IgG	Sheep	HRP	NA9310	Amersham

## Data Availability

The guarantors for the content of the article are D.B. and G.P. The data presented in this study are available within the article and upon request from the corresponding author.

## Supplementary Materials

The supporting information can be downloaded at: https://www.mdpi.com/article/10.3390/ijms231911070/s1.
